# Antibacterial Thermosensitive Silver–Hydrogel Nanocomposite Improves Wound Healing

**DOI:** 10.3390/gels9070542

**Published:** 2023-07-04

**Authors:** Nafise Amiri, Sahand Ghaffari, Ida Hassanpour, Taesik Chae, Reza Jalili, Ruhangiz Taghi Kilani, Frank Ko, Aziz Ghahary, Dirk Lange

**Affiliations:** 1 Professional Fire Fighters’ Burn and Wound Healing Research Laboratory, Division of Plastic Surgery, Department of Surgery, University of British Columbia, Vancouver, BC V5Z 1M9, Canadaida.hassanpour@gmail.com (I.H.); ghaharya@yahoo.com (A.G.); 2 ICORD and Department of Pathology & Laboratory Medicine, University of British Columbia, Vancouver, BC V5Z 1M9, Canada; 3 The Stone Centre at Vancouver General Hospital, Department of Urologic Sciences, University of British Columbia, Vancouver, BC V5Z 1M9, Canada; 4 Department of Materials Engineering, University of British Columbia, Vancouver, BC V6T 1Z4, Canada; 5 Aspect Biosystems, Vancouver, BC V6P 6P2, Canada; rbjalili@gmail.com

**Keywords:** hydrogel nanocomposite, silver nanoparticle, wound healing, antibacterial wound dressing

## Abstract

Bacterial infection and poor cell recruitment are among the main factors that prolong wound healing. To address this, a strategy is required that can prevent infection while promoting tissue repair. Here, we have created a silver nanoparticle-based hydrogel composite that is antibacterial and provides nutrients for cell growth, while filling cavities of various geometries in wounds that are difficult to reach with other dressings. Silver nanoparticles (AgNPs) were synthesized by chemical reduction and characterized using transmission electron microscopy (TEM), dynamic light scattering (DLS), and inductively coupled plasma-mass spectroscopy (ICP-MS). Using varying concentrations of AgNPs (200, 400, and 600 ppm), several collagen-based silver–hydrogel nanocomposite candidates were generated. The impact of these candidates on wound healing was assessed in a rat splinted wound model, while their ability to prevent wound infection from a contaminated surface was assessed using a rat subcutaneous infection model. Biocompatibility was assessed using the standard MTT assay and in vivo histological analyses. Synthesized AgNPs were spherical and stable, and while hydrogel alone did not have any antibacterial effect, AgNP–hydrogel composites showed significant antibacterial activity both in vitro and in vivo. Wound healing was found to be accelerated with AgNP–hydrogel composite treatment, and no negative effects were observed compared to the control group. The formulations were non-cytotoxic and did not differ significantly in hematological and biochemical factors from the control group in the in vivo study. By presenting promising antibacterial and wound healing activities, silver–hydrogel nanocomposite offers a safe therapeutic option that can be used as a functional scaffold for an acceleration of wound healing.

## 1. Introduction

Chronic wounds affect millions of patients all around the world and contribute significantly to their morbidity and mortality [1,2,3]. Among the many factors that may limit effective wound healing in patients with chronic wounds, bacterial infection and biofilm formation and poor cell recruitment are primary causes that contribute to prolonged healing [3,4,5,6,7]. Therefore, a strategy that aims to prevent bacterial infection within the wound, while simultaneously providing structural scaffolding that promotes endogenous tissue repair, would be of great interest.

Hydrogels are promising candidates for wound dressings due to their extraordinary properties such as biocompatibility, tunable mechanical properties, and good permeability [8]. The high water content and their ability to keep the wound environment moist is also proven to facilitate healing [9]. In addition to their inherent properties, other functional aspect such as enhanced cell attachment, angiogenesis, and antibacterial activity could be integrated into hydrogels to provide superior functionality for treatment of chronic wounds [10]. A number of natural and synthetic polymeric materials are in use to produce hydrogel dressings. Our group has previously reported the injectable hydrogel composed of cross-linked bovine collagen type I and chondroitin sulfate, supplemented with polyvinyl alcohol (PVA), and it contains optimum concentration of necessary amino acids, vitamins, and minerals required for cell growth and proliferation [11]. 

A previous body of work published by our group describes the synthesis and complete characterization of a novel hydrogel-based scaffold shown to be biocompatible and result in improved healing of open wounds in a mouse wound healing model [12,13,14]. Given the potential for the bacterial contamination of wounds to lead to significantly delayed healing due to infection, we decided to develop the characteristics of the scaffold to include antibacterial activity via the incorporation of silver nanoparticles (AgNPs) into the hydrogel. Silver is a well-known antimicrobial agent already used widely throughout medicine including in wound care management [15,16]. While effective, these therapies are associated with limitations including toxicity and skin discoloration [17,18]. AgNPs have shown the potential to circumvent these limitations and have gained considerable attention in wound bioburden reduction and as anti-inflammation agents as they can release Ag^+^ ions at a greater rate than bulk silver, by virtue of their large specific surface area [19,20]. In this work, we developed a new Ag–hydrogel nanocomposite, initially synthesized using two non-toxic reducing agents. Following extensive characterization, we reconstituted the freeze-dried collagen–GAG hydrogel in a AgNP colloidal solution containing varying concentrations of silver to identify the best Ag-containing hydrogel nanocomposite. Overall, we found the Ag–hydrogel nanocomposites to exhibit very effective bactericidal activity against both Gram-negative and Gram-positive bacteria in the absence of cytotoxicity. Furthermore, accelerated wound closure and faster re-epithelialization were observed when the efficacy and safety of the Ag–hydrogel nanocomposites were assessed in vivo.

## 2. Results and Discussion

Bacterial infection and poor cell recruitment are two primary factors that can significantly impede the process of wound healing in patients with chronic ulcers. As a result, finding innovative approaches that effectively address these issues and simultaneously facilitate endogenous tissue repair through structural scaffolding holds immense promise. Such a novel strategy would not only prevent bacterial infection within the wound but also promote the natural healing process, leading to expedited and improved wound healing outcomes. Previously, we developed a thermosensitive collagen–GAG-based hydrogel that is liquid at low temperatures (<4 °C), solidifies within 10 to 20 min of application to the wound sites, and contains all the amino acids, vitamins, and minerals required for cell growth. Being in a liquid form when applied to the wound, it fills up all the cavities and void areas seen in many different skin defects, injuries, and non-healing wounds. To improve the current product such that it would be able to manage infection and protect wounds from bacterial contamination, we used AgNPs to add antibacterial activity to the hydrogel. For this purpose, first we synthesized and characterized AgNPs. Next, lyophilized collagen–GAG hydrogel powder was reconstituted in AgNPs colloidal solution with different concentrations of AgNPs up to 600 ppm to form Ag–hydrogel nanocomposite.

AgNPs were synthesized by chemical reduction method and then characterized. The synthetic pathway involved using two new reducing agents that are safe and non-toxic, which allowed for the synthesis of Ag nanoparticles with narrow and unimodal size distribution.

**Figure 1:**
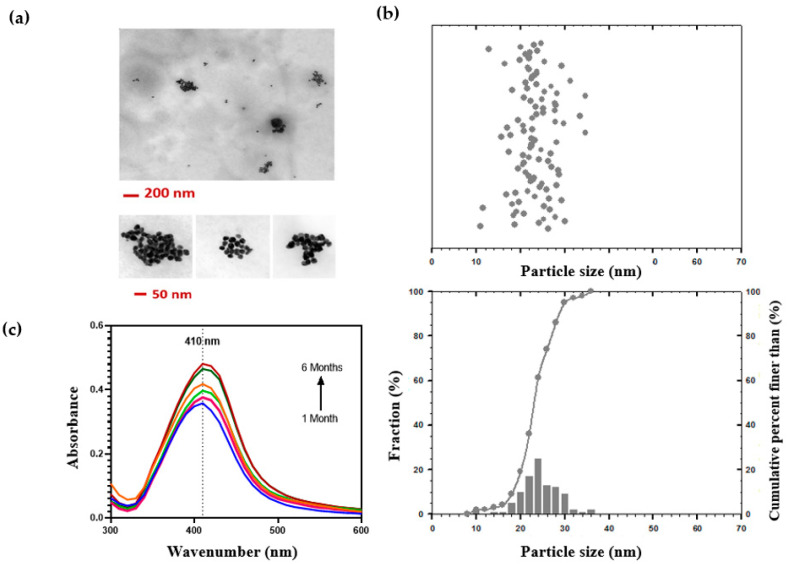
Characterization of synthesized silver nanoparticles. (**a**) TEM images. (**b**) Particle size distribution processed by ImageJ. (**c**) UV–Visible absorption spectra of synthesized silver nanoparticles after storage at 4 °C for 6 months, showing a stable absorption band at 410 nm for 6 months. Each month’s spectrum is displayed in a range of colors, transitioning from blue (1st month) to red (6th month).

Physicochemical properties of AgNPs are key factors in antibacterial activity and toxicity. We used dynamic light scattering (DLS) and transmission electron microscopy (TEM) to measure size, dispersity, and zeta potential of nanoparticles. The TEM images showed nanoparticles had a spherical shape with an average diameter of 23.34 ± 4.37 nm (Figure 1a,b). DLS identified nanoparticles with hydrodynamic diameter in the range of 31.17 ± 0.20 nm (Supplementary Data S1) and zeta potential in the range of −28.10 ± 1.44 mV, which indicated good physical stability of the dispersion due to electrostatic repulsion of individual particles. DLS was also used to evaluate the uniformity of nanoparticle dispersion by measuring Poly Dispersity Index (PDI). The PDI values below 0.2 indicate a narrow size distribution [21]. Here, the PDI of 0.192 ± 0.001 (Supplementary Data S1) shows a narrow unimodal distribution in size for the synthesized silver nanoparticles.

These results were further confirmed by UV–Vis spectroscopy, which is the most widely used technique for structural characterization of AgNPs. The optical properties of spherical AgNPs are highly dependent on nanoparticle diameter and change when particles aggregate. The absorption spectrum of AgNPs solution immediately after synthesis displayed a symmetrical strong absorption peak, associated with the Surface Plasmon Resonance (SPR), at 410 nm. This finding is consistent with the previous studies, showing that the SPR peak for the spherical AgNPs normally appears between 410 and 480 nm. In addition, the absence of any additional peak and the symmetrical shape of the plasmon band were a good indicator of its monodispersity. As for stability assessment over time, the same characteristic absorption band was detected during 6-month storage at 4 °C (Figure 1c), displaying the high dispersion stability of AgNPs solution despite long storage.

**Figure 2:**
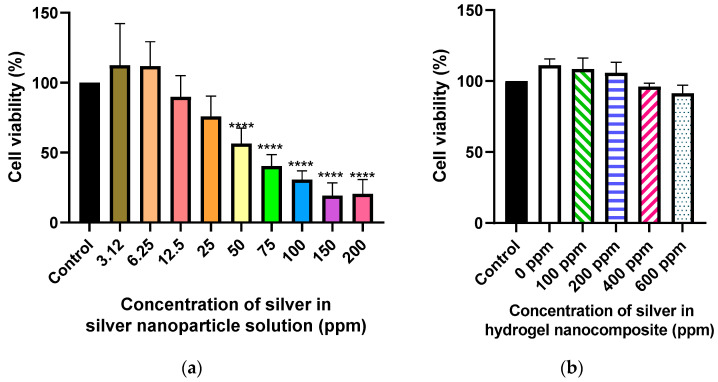
Cytotoxicity. (**a**) Cell viability (%) of human dermal fibroblast cells incubated in direct contact with MeshFill alone and loaded with different concentrations of AgNPs. (**b**) Cell viability (%) of human dermal fibroblast cells incubated with an extract of Ag–hydrogel nanocomposite with different concentrations of silver (0, 100, 200, 400, and 600 ppm). The cytotoxicity was evaluated based on the International Organization for Standardization 109993-5 (**** *p* < 0.0001).

The toxicity of AgNPs has been broadly evaluated throughout the literature, with the overall results being quite variable. The main reason for this variation is that the toxicity of AgNPs is highly dependent on their size and shape, the degree of viscosity, and water solubility of the nanoparticle formulation as well as the extent of functionalization of the ligands binding to nanoparticles surface, which can dictate protein adsorption [22,23]. Generally, compared to silver ions, AgNPs show less toxicity both on human dermal fibroblasts and human dermal keratinocytes [24]. When investigating the interaction of human dermal fibroblasts with AgNPs of different sizes, Avalos et al. showed smaller particles were much more toxic than the larger AgNPs [25]. In our study, the MTT assay on human dermal fibroblast cultured with different concentrations of AgNPs solution showed dose-dependent toxicity with an IC_50_ value of 64 ppm (Figure 2a). This result was very close to results obtained by other investigators for AgNPs of similar size [26,27]. Furthermore, we applied the ISO standard method to investigate the cytocompatibility of the formulation [28]. Interestingly, when combining two systems consisting of AgNPs and collagen hydrogel, the resulting nanocomposite did not have cytotoxic effects (Figure 2b). Both hydrogel alone and Ag–hydrogel nanocomposite groups showed high cell viability (>80%), suggesting that the formulations are biocompatible. These results suggest that the hydrogel controls the release of AgNPs, minimizing the cytotoxic effect on the cells. These findings support the potential application of Ag–hydrogel nanocomposites as hydrogel dressings for wound management.

AgNPs have broad-spectrum antibacterial activity against different bacterial species including Methicillin-resistant *Staphylococcus aureus* (*MRSA*) and *Pseudomonas aeruginosa* (*PA*), which are the most common bacterial species responsible for wound infections. The possible mechanism of the AgNPs’ antibacterial activity is attributable to a combined effect of both AgNPs and Ag ions including attachment and disruption of the bacterial membrane, damage of intracellular biomolecules and structures, and the induction of oxidative stress with generation of reactive oxygen species (ROS) and free radicals [29].

In our study, the MICs of AgNPs against *MRSA* and *PA* were found to be 79.18 µg/mL and 6.29 ± 0.690 µg/mL, respectively. These may be different from values reported in the literature, mainly due to differences in the specific bacterial strains used in a given study. In addition, a direct comparison is not possible as characteristics specific to the AgNP used in a given study that impact activity may differ, including variation in the particle’s physicochemical properties such as size, shape, crystallinity, surface chemistry, and capping agent [30]. While smaller particles exhibited stronger antibacterial efficacy compared to those with larger sizes, 20 nm AgNPs showed an MIC of 90 μg/mL against *S. aureus*, while that for *PA* was closer to those reported earlier [31,32,33]. Similar to our observation, the efficacy of AgNPs of a given size differs between Gram-positive versus Gram-negative bacteria, as indicated by varying MIC values. Interestingly Gram-positive bacteria such as *S. aureus* are more resistant to AgNPs compared to Gram-negative bacteria such as *P. aeruginosa*, which may be the result of differences in the overall composition between these bacterial groups, resulting in differences in overall thickness and outer membrane surface charges [29,34]. For instance, Gram-negative bacterial species have a thinner peptidoglycan layer, resulting in easier penetration of AgNPs into these bacteria and greater susceptibility [35].

**Figure 3:**
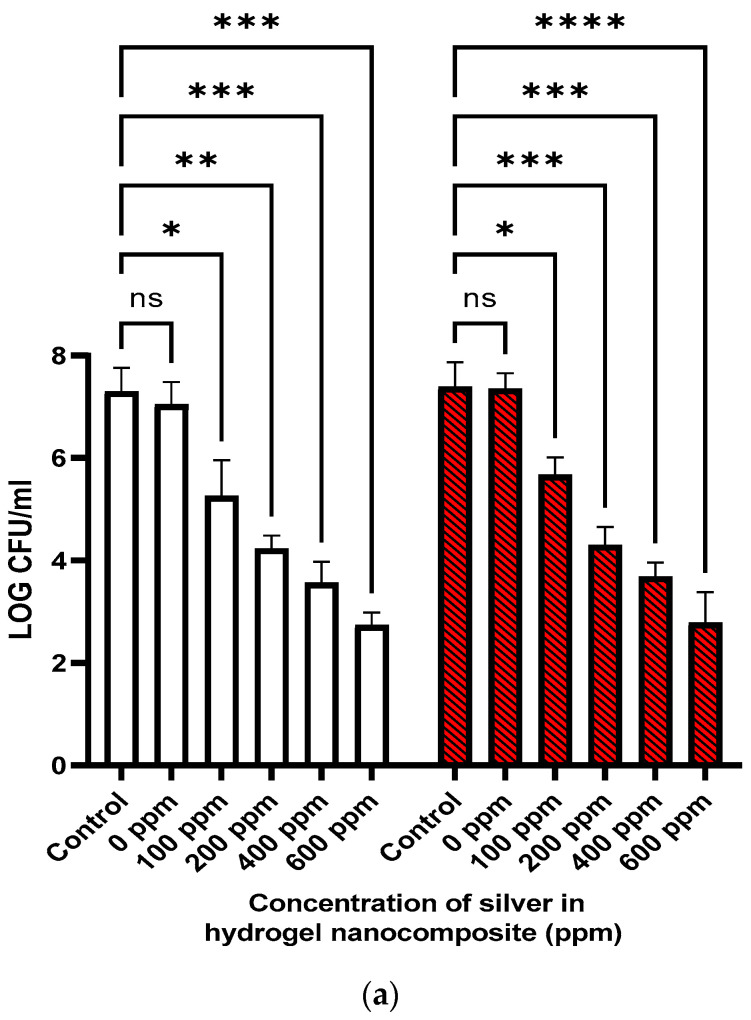

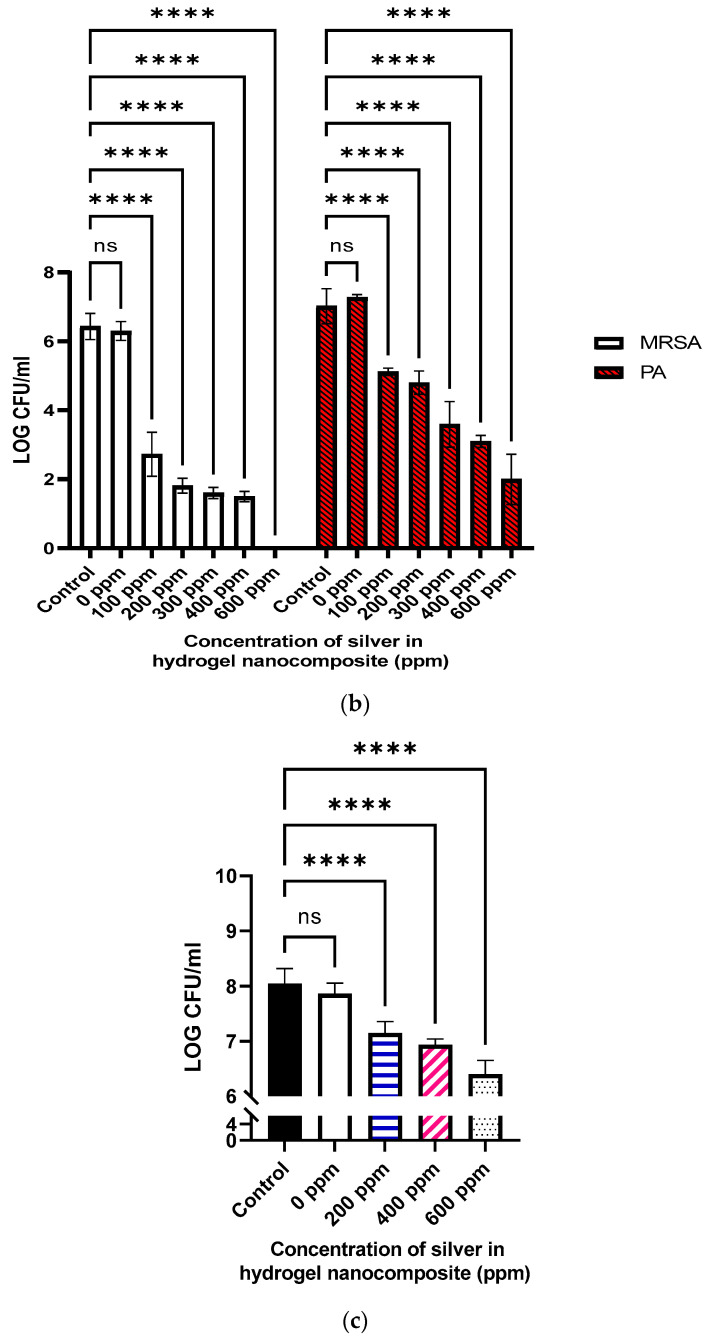

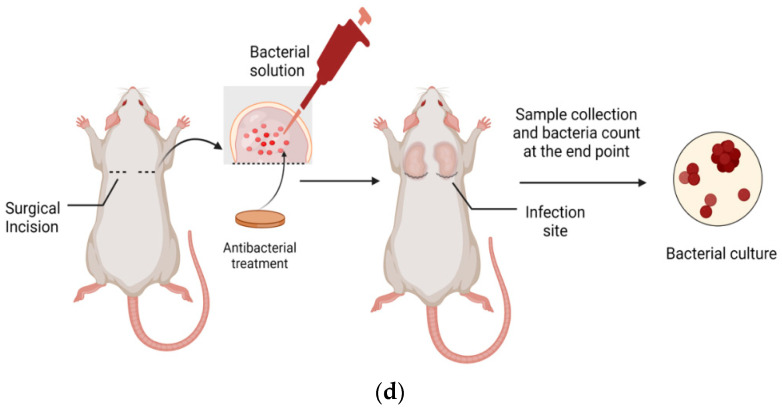
Antibacterial activity. (**a**) Ag–hydrogel nanocomposite with different concentrations of AgNPs (0, 100, 200, 400, and 600 ppm) was placed in bacterial solution to be in direct contact with two common wound-infecting bacteria, *Methicillin-resistant Staphylococcus aureus* (*MRSA*) (Gram-positive) and *Pseudomonas aeruginosa* (*PA*) (Gram-negative). After 24 h, small aliquots of the bacterial solution were removed and microorganisms in it were enumerated. Bacterial solution alone was considered as a control. In control and hydrogel alone (0 ppm), the significant increase in the number of bacteria was observed in solution after 24 h, while AgNPs significantly inhibited the growth of bacteria in Ag–hydrogel nanocomposite groups. (**b**) The effect of Ag–hydrogel nanocomposites in preventing bacteria penetration into the hydrogel. Sterile Titanium (Ti) wire disc was cast inside the hydrogels. The samples were placed in bacterial solution to be in direct contact with two common wound-infecting bacteria. After 24 h, Ti disc was harvested from inside of the hydrogels, and microorganisms on the surface was enumerated. Ti disc alone in bacterial solution was considered as a control. After 24 h, all Ag–hydrogel nanocomposite groups significantly inhibited penetration of bacteria inside the gel and growth of bacteria on Ti wire surface. (**c**) In vivo antibacterial activity. Antibacterial activity of formulations was evaluated in subcutaneous infected wound on the dorsal area of rat against *Methicillin-resistant Staphylococcus aureus* (*MRSA*). While there was no difference between untreated (control) and hydrogel alone (0 ppm), a significant decrease was observed in CFU of bacteria in all Ag–hydrogel nanocomposite groups. (**d**) Schematic images of in vivo antibacterial study. (* *p* < 0.05, ** *p* < 0.01, *** *p* < 0.001, **** *p* < 0.0001).

Given that the AgNPs need to retain their activity when incorporated into the actual hydrogel, we verified the antibacterial activity of the Ag–hydrogel nanocomposite against *MRSA* and *PA*. As shown in Figure 3a, exposure of bacteria to hydrogel alone did not result in a reduction in bacterial numbers, while exposure to all Ag-containing hydrogels significantly inhibited bacterial growth in a dose-dependent fashion. In Ag–hydrogel nanocomposite groups, the drop in the number of bacteria increased with increasing Ag concentration, and maximum reduction was observed for the 600 ppm group against both pathogens that resulted in a 4.20 ± 0.33 log reduction in *PA* compared to only 1.71 ± 0.35 for the 100 ppm sample. Similarly, the log reduction against *MRSA* was 4.56 ± 0.26 for the 600 ppm sample compared to 2.04 ± 0.69 for the 100 ppm sample.

To assess efficacy of the cast formulations in a more realistic environment, we determined the antimicrobial activity of the cast formulations in a subcutaneous implant infection model. For this, the cast formulations were implanted into subcutaneous pockets on the backs of Sprague–Dawley rats followed by the addition of *MRSA* (10^6^ CFU/mL) prior to suturing the implantation site to induce an infection (Figure 3d). Overall, we found a significant reduction in bacterial numbers on day 4 post-infection in the infected pockets containing Ag–hydrogel samples compared to those that only contained hydrogel samples (Figure 3c).

Next, we assessed the potential for the hydrogel to prevent the colonization of a substrate surface that is encased by the hydrogel itself as this most closely mimics the scenario where a hydrogel-treated surface may be challenged with a higher load of contamination bacteria. For this, we cast Ti discs inside hydrogel or Ag–hydrogel and exposed them to a suspension containing 10^6^ *MRSA.* The hydrogel or Ag–hydrogel containing the Ti discs were then implanted into subcutaneous pockets of rats as described above, and the animals were recovered for 4 days. Overall, Ag–hydrogel was found to significantly reduce the bacterial penetration through the hydrogel and onto the Ti disc surface compared to Ti discs encased in hydrogel only or without hydrogel. The greatest reduction (log 6.43 ± 0.38) was found with Ag–hydrogel containing 600 ppm Ag that completely prevented the colonization of the underlying Ti discs with *MRSA*.

Interestingly, while the MIC of AgNPs was lower for *PA*, AgNP–hydrogel composite showed better antibacterial activity against *MRSA* in the penetration test. This could be because *PA* is capable of two distinct types of surface-specific motilities, twitching and swarming [36].

These results confirm that in addition to high cytocompatibility, the AgNP–hydrogel composite exhibited strong antibacterial activity. Such behavior has been reported previously in different synthetic and natural polymers including PVA, PVP, gelatin, and alginate used to prepare antimicrobial Ag–hydrogel nanosystems. Travan et al. described synthesis and stabilization of AgNPs in a chitosan-derived polysaccharide solution and studied the cytotoxicity and antibacterial properties in both solution and within a 3D hydrogel structure. Antimicrobial results showed that the nanocomposite system displays a very effective bactericidal activity toward both Gram-positive and Gram-negative bacteria. However, the hydrogel does not show any cytotoxic effect towards the three different eukaryotic cell lines including mouse fibroblast-like (NIH-3T3) cells. They suggested that the nanoparticles, immobilized in the hydrogel matrix, can exert their antimicrobial activity by simple contact with the bacterial membrane, while entrapping AgNPs in hydrogel could prevent them from diffusing into the surrounding environment and making them available for cells to be taken up [37].

Chronic non-healing wounds have a significant impact on numerous patients every year, significantly contributing to their morbidity and mortality. Complications and delayed wound healing are often a result of bacterial infection. Thus, wound dressings with advanced antibacterial activity are of great interest within wound care management. Antibiotic-loaded dressing with controlled release activity can provide antibacterial activity while avoiding the exposure of the healing tissue to toxic concentrations of active agent [38].

To assess the effect of Ag–hydrogel on the wound healing process, we utilized the rodent excisional wound healing model. Considering that the rodent skin is mobile, wound closure for the most part takes place via contraction. To overcome this shortcoming, we used an excisional splinting model in rats. In this model, a splinting ring is tightly attached to the skin around the wound to prevent skin contraction, forcing the wound to heal through granulation and re-epithelialization, a process similar to that occurring in humans.

**Figure 4:**
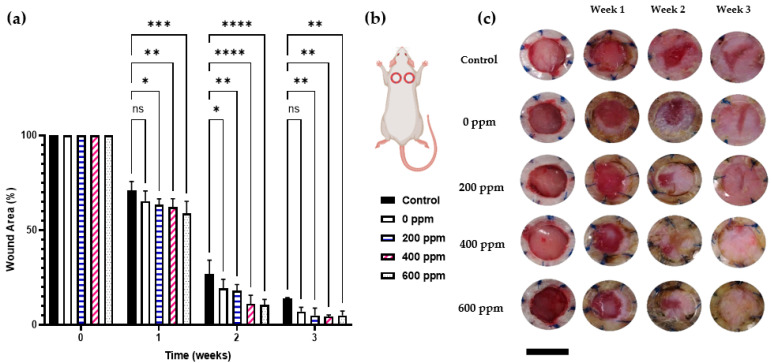
Wound healing study. (**a**) Average wound area measured using Image J^®^ software and plotted as relative % of original wound. (**b**) Schematic image of the location of splinted wounds on back of rat. (**c**) Representative serial digital images of top view of full-thickness splinted wounds on dorsal region of rats either kept untreated (control) or treated with silver–hydrogel nanocomposites with different concentration of silver (0, 100, 200, 400, and 600 ppm) at week 1, 2, and 3 (* *p* < 0.05, ** *p* < 0.01, *** *p* < 0.001, **** *p* < 0.0001).

**Figure 5:**
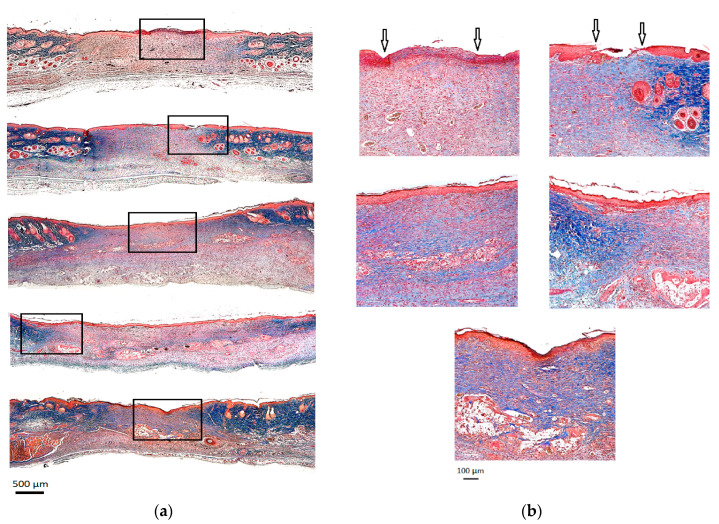
Histological analysis by Masson’s Trichrome staining. (**a**) The whole wound at 2× magnification. (**b**) The wound at 10× magnification. Black arrows demonstrate incomplete epithelialization.

As shown in Figure 4, wounds exposed to all Ag–hydrogel nanocomposite groups consistently closed faster than both hydrogel alone and control groups, and the original wound area in rats treated with Ag-containing group was significantly smaller at weeks 1, 2, and 3 post-wounding (Figure 4a,b). That said, using a two-way ANOVA, statistically significant differences in the wound healing process were observed in wounds treated with either hydrogel or Ag–hydrogel nanocomposite groups compared to control. Having confirmed that both hydrogel or Ag–hydrogel nanocomposite treatments accelerated the wound healing process from clinical observation, we therefore wondered whether they could provide a favorable microenvironment to speed up the restoration of epidermal and dermal architecture. To investigate this, histological analysis was performed to assess the effect of treatments on skin wound healing in the microstructure. The Trichrome staining of wound site harvested at the end of study (week 3) is shown in Figure 5. Compared to control, wound re-epithelialization was remarkably enhanced after treatment with all Ag–hydrogel nanocomposites. Therefore, the clinical and histological observations of the in vivo study revealed accelerated re-epithelization and better wound contraction with AgNP–hydrogel composites. This result was in agreement with other studies evaluating the wound healing efficacy of biomaterials containing AgNPs.

In addition to antibacterial activity, AgNPs play an active role in wound healing. Previous studies have shown that AgNPs promote the migration of fibroblasts and stimulate the differentiation of fibroblasts to myoblasts, which speed up the wound contraction and promote the healing process [39]. AgNPs also improve proliferation and migration of keratinocytes from the edge to the center of the wound and trigger the differentiation and maturation of keratinocytes, thus promoting wound contraction [40]. Moreover, the anti-inflammatory property of AgNPs is another mechanism that supports the wound healing process by reducing the level of proinflammatory cytokines or decreasing mast cell infiltration [41].

## 3. Conclusions

In this manuscript, we discuss the synthesis and characterization of collagen-based Ag–hydrogel nanocomposites with excellent wound healing and antibacterial properties. The findings of this study address some of the major shortcomings of currently available wound healing products including bacterial biofilm formation, infection development, and resultant impaired wound healing. The incorporation of AgNPs into an already highly effective hydrogel scaffold resulted in significant antibacterial and pro-wound healing activities including accelerated re-epithelization and better wound healing without cytotoxic effects. Given this, the Ag–hydrogel nanocomposite developed herein has significant potential to be a safe therapeutic option as a functional scaffold for the acceleration of wound healing.

## 4. Methods

### 4.1. Ag–Hydrogel Nanocomposite

#### 4.1.1. Silver Nanoparticle Synthesis and Ag–Hydrogel Nanocomposite Fabrication

Silver nanoparticles (AgNPs) were synthesized by chemical reduction method [42,43]. Silver nitrate (AgNO_3_) as a source of silver was reduced by trisodium citrate and glucose as reducing agents. A 1 mL volume of 30 mM trisodium citrate was added to 100 mL of distilled deionized water (DDW) under constant stirring and heated up. As the temperature reached 95 °C, 2 mL of 5 mM AgNO_3_ and 50 mM glucose were added to the solution drop by drop. The reaction mixture was stirred vigorously on a magnetic stirrer and heated until the solution color turned light yellow. The resulting AgNPs solution was kept in dark at 4 °C for future use. The solution was further centrifuged (10,000 rpm for 15 min) and resuspended in DDW to prepare aqueous AgNPs with different concentrations. To form Ag–hydrogel nanocomposite, lyophilized collagen–GAG hydrogel powder was reconstituted in AgNPs aqueous solution with different concentrations.

#### 4.1.2. Silver Nanoparticle Characterization

Synthesized AgNPs were visualized using transmission electron microscopy (TEM, H7600, Hitachi, Tokyo, Japan). AgNPs solution was diluted with DDW. Then, a drop of the solution was placed on a TEM grid and dried at room temperature. The AgNPs were viewed at an operating voltage of 80 kV, and the diameter of 100 particles was measured using ImagJ to assess the particle size and distribution. The particle size distribution of AgNPs was also confirmed using dynamic light scattering (DLS, NanoBrook Omni, Brookhaven Instruments, Holtsville, NY, USA) before proceeding to Ag–hydrogel nanocomposite preparation. The AgNPs solution was diluted with DDW and transferred to a square plastic cuvette. Solid-state laser (35 mW red diode) with a wavelength of 640 nm was used, and the scattering detection angle was chosen to be 90° [44]. Another square plastic cuvette was prepared to measure the zeta potential of AgNPs by phase analysis light scattering (PALS) to assess the colloidal stability of the solution. The AgNPs solution concentration was measured using Inductively coupled plasma-mass spectroscopy (ICP-MS). The absorbance spectrum of the colloidal sample was obtained in the range of 300–700 nm using a UV–Vis spectrometer Shimadzu-UV. The contents, preparation, and properties of collagen–GAG hydrogel have been described in detail previously [11].

### 4.2. In Vitro Cytocompatibility

#### 4.2.1. Silver Nanoparticle Cell Viability Assay

The cytotoxic effect of synthesized silver nanoparticles was measured using MTT assay. Human dermal fibroblasts (HDF) cells were seeded in a 96-well tissue culture plate at a density of 1.5 × 10^4^ cells/well with a pre-warmed fresh medium (DMEM containing 10% fetal bovine serum and 1% penicillin/streptomycin) and incubated at 37 °C in an incubator supplied with 5% CO_2_ for 24 h. After that, the cells were treated with different concentrations of silver nanoparticles (0, 50, 100, and 200 ppm) and incubated for another 24 h. Next, the test solutions were removed, 100 μL of MTT-containing medium (1 mg/mL) was added to each well and further incubated for 4 h. Then, the supernatants were removed and replaced with 100 μL DMSO to solubilize the resulting purple formazan crystals formed in living cells. The relative cell viability (%) was calculated based on the absorbance at 570 nm measured with a microplate reader (BioTek, Santa Clara, CA, USA) according to the following formula:

Cell viability (%) = (optical density of treated cells/optical density of control cells) × 100(1)

#### 4.2.2. Silver–Hydrogel Nanocomposite Cytocompatibility

The cytocompatibility of Ag–hydrogel nanocomposite containing different concentrations of AgNPs (0, 100, 200, 400, and 600 ppm) was evaluated based on the International Organization for Standardization 109993-5 using extract analysis. In brief, formulations were extracted in culture medium at 37 °C for 24 h, and the extracts were collected for cell culture. Human dermal fibroblasts (HDF) cells were seeded at a density of 1.5 × 10^4^ cells per well of a 96-well plate with a DMEM-high glucose supplemented with 10% FBS and a 1% penicillin/streptomycin solution and incubated at 37 °C and supplied with 5% CO_2_. After 24 h of incubation, the culture medium was removed, each well was washed with phosphate-buffered saline (PBS) three times, and then 100 µL of extracts was added per well. Cell proliferation and viability was determined using MTT assay after 24 h of exposure.

### 4.3. Antibacterial Activity

#### 4.3.1. Minimum Inhibitory Concentration (MIC) of AgNPs Solution

A standard broth microdilution method was used to determine the minimal inhibitory concentrations (MIC) of synthesized AgNPs against American type culture collection (ATCC) strains of Methicillin-resistant Staphylococcus aureus (MRSA-USA300) (Gram-positive) and Pseudomonas aeruginosa (PAO1-#166.lux) (Gram-negative). In brief, a total of eight serially diluted samples were used, starting with a 200 ppm stock solution of AgNPs, two-fold diluted in ddH2O (100 µL total volume) seven additional times. The bacterial isolates were cultured and sub-cultured in LB as the growth media under aerobic conditions at 37 °C, 48 and 24 h prior to all experiments. A starting colony-forming units (CFU) of 10^5^–10^6^ of each bacterial strain were inoculated with the serially diluted AgNPs in 96-well plates and incubated for 24 h. After that, viable counting colonies were enumerated using serial dilution of a 10 µL aliquot from each sample in PBS followed by growth on agar Petri dish by incubating for 14–18 h at 37 °C, according to the following equation:

CFU/mL = [(# of countable colonies) × (1/dilution factor) × 1000 µL/mL]/(10 µL)(2)

#### 4.3.2. Antibacterial Activity of Ag–Hydrogel Nanocomposite Formulations

Two different methods were applied to evaluate the antibacterial activity of the formulations in vitro. In the first method, the disc-shaped casted hydrogels were directly placed in bacterial solution to be in direct contact with two common wound infecting bacteria, *MRSA* and *PA*. Briefly, 200 µL/well of hydrogel was added to 48–well plate and kept at room temperature for 10–15 min to cast. Then, microbial culture containing 10^6^ CFU/mL of bacteria was added to the well and then placed in 37 °C incubator. After 24 h, small aliquots of the bacterial solution were removed and microorganisms in it were enumerated as stated above.

In the second method, the effect of Ag–hydrogel nanocomposites in preventing bacteria penetration into the hydrogel was evaluated. To do so, a sterile disc-shaped Titanium (Ti) wire was placed inside the AgNP–hydrogels prior to casting. Similar to previous method, the hydrogel was placed in bacterial solution after casting. The Ti disc was subsequently harvested, and number of bacteria infiltrated into the hydrogel and attached to the Ti disc was counted after 24 h incubation at 37 °C.

### 4.4. In Vivo Study

#### 4.4.1. Experimental Animals

Healthy female Sprague–Dawley (SD) rats (200–300 g, aged 16–24 weeks old) were obtained from Charles River Laboratories. Animal care and all experimental procedures were performed according to the Guide for the Care and Use of Laboratory Animals of the University of British Columbia Animal Care Committee. The clearance to conduct this study was provided by the University of British Columbia Committee of Animals and Ethics (protocol approval ID: A17-0292 for antibacterial study and A18-0366 for wound healing study). The rats were maintained with water and standard laboratory chow ad libitum for 1 week prior to the experiment to acclimatize to the laboratory environment in a temperature-controlled room. The animals were randomly divided into study groups and were individually anesthetized using 2% isoflurane in oxygen (flow rate of 1.0 L/min) and kept under anesthesia throughout the procedure.

#### 4.4.2. In Vivo Antibacterial Study

To determine the efficacy of Ag–hydrogel nanocomposite formulations on bacterial growth inhibition in an in vivo setting, we used a subcutaneous infection wound model that was recently used for evaluation of antibacterial activity of different biomaterials using rats [45]. After fur removal with electric clipper, an 8 mm incision was made on either side of the median line on the dorsal aspect of the animal. A subcutaneous pocket was formed by blunt dissection technique (using a sterile hemostat) large enough to insert a 1 cm × 0.5 cm cast hydrogel that was either bare or loaded with AgNPs. Infection was induced by inoculating 10^6^ MRSA inside each pocket. Following implantation, the incisions were closed with absorbable sutures. On day 4 post-surgical implant, animals were sacrificed and the whole pockets and surrounding tissue were harvested. Harvested tissues were then homogenized using a Precellys 24 tissue homogenizer, and the number of bacteria were quantified by serial dilution in PBS, followed by plating on agar and CFU counting in 14–18 h.

#### 4.4.3. In Vivo Wound Healing Study

The rat excisional wound splinting model was used to determine the efficacy of Ag–hydrogel nanocomposite in wound healing. Following aseptic surgical techniques, two full-thickness excisional wounds with 8 mm circular diameter were created bilaterally on either side of the vertebral column of each rat. To overcome the problem of wound contracture in rodent wounds, silicone splints with an inner and outer diameter of 10 and 14 mm were secured on the edges of the wound. Five experimental groups were tested with either: (1) clean untreated wounds or (2) clean wounds filled with CG hydrogel alone; (3–5) clean wounds filled with Ag–hydrogel nanocomposite containing 200, 400, and 600 ppm of Ag, respectively. The wound healing process was monitored every 3 days for up to 3 weeks.

##### Wound Surface Area Calculation

Digital images of the wound area were captured on different time points. The images were processed with ImageJ^®^ software (Image J 1.4, National Institutes of Health (NIH), Bethesda, MD, USA) for surface area changes. Total reduction in the wound size was calculated by dividing the surface area at each time point to that of the original wound using the following equation:

(3) $$\mathrm{Total}\;\mathrm{Wound}\;\mathrm{Closure}\;(\%)=100-[\frac{Wound\;surface\;area}{Original\;wound\;surface\;area}\times 100]$$

##### Histological Analysis

At the end point of the study, all wound sites were harvested for histological evaluation. Tissue samples were fixed in 10% neutral buffered formalin solution (Sigma-Aldrich, St. Louis, MO, USA) for 24 h before being processed and embedded in paraffin blocks. Samples were sectioned at 5 µm thickness and stained using Masson’s Trichrome stain. Briefly, slides were incubated in Bouin’s solution for 1 h at 60 °C. Then, rinsing slides were stained with Weigert’s Hematoxylin solution (Sigma-Aldrich) followed by Biebrich Scarlet-Acid Fuchsin, phosphomolybdic–phosphotungstic acid, aniline blue, and 1% acetic acid solution. Collagen appears blue, nuclei black, and cytoplasm red.

##### Hematology Analysis and Biochemical Assay

To examine the safety and evidence of systemic inflammatory response, blood cell count and biochemical assay were conducted at the end of animal study. Blood samples were obtained by cardiac puncture. Separate portions of blood were transferred to tubes containing either lithium heparin or trisodium EDTA as anticoagulants. The whole blood was used to count total red blood cells (RBC), white blood cells (WBC), platelets, and hemoglobin (or hematocrit). The blood serum was processed in a VETSCAN Analyzer (Abaxis Inc., Sunnyvale, CA, USA) for the analysis of the following parameters: total protein (TP), alkaline phosphatase (ALP), alanine transaminase (ALT), glucose (GLC), blood urea nitrogen (BUN), and creatinine (CR).

### 4.5. Statistical Analysis

All data were presented as the mean ± standard deviation of three independent experiments. Data were subjected to statistical analysis using paired Student’s *t*-test and one-way or two-way analysis of variance (ANOVA) with GraphPad Prism 9.0.0 (121) (GraphPad Software Inc., San Diego, CA, USA). Post hoc test was performed using the Dunnett test. Mean values were considered to be statistically significant at *p* < 0.05 (*), *p* < 0.01 (**), *p* < 0.001 (***).

## Data Availability

The data that support the findings of this study are available from the corresponding author upon request.

## Supplementary Materials

The following supporting information can be downloaded at: https://www.mdpi.com/article/10.3390/gels9070542/s1, Supplementary Data S1: Dynamic Light Scattering (DLS) graphs and size measurement of silver nanoparticles. The particle size distributions (MSD: multimodal size distribution, LND: lognormal size distribution) are expressed using intensity-based and numerical-based calculations.
