# Supplementary material for: Antibacterial Thermosensitive Silver–Hydrogel Nanocomposite Improves Wound Healing
Source: Gels. 2023 Jul 4;9(7):542. doi: 10.3390/gels9070542 (PMC10379397; doi:10.3390/gels9070542)
Supplement: Supplementary file 1 [file gels-09-00542-s001.zip › gels-2459356-supplementary.pdf]

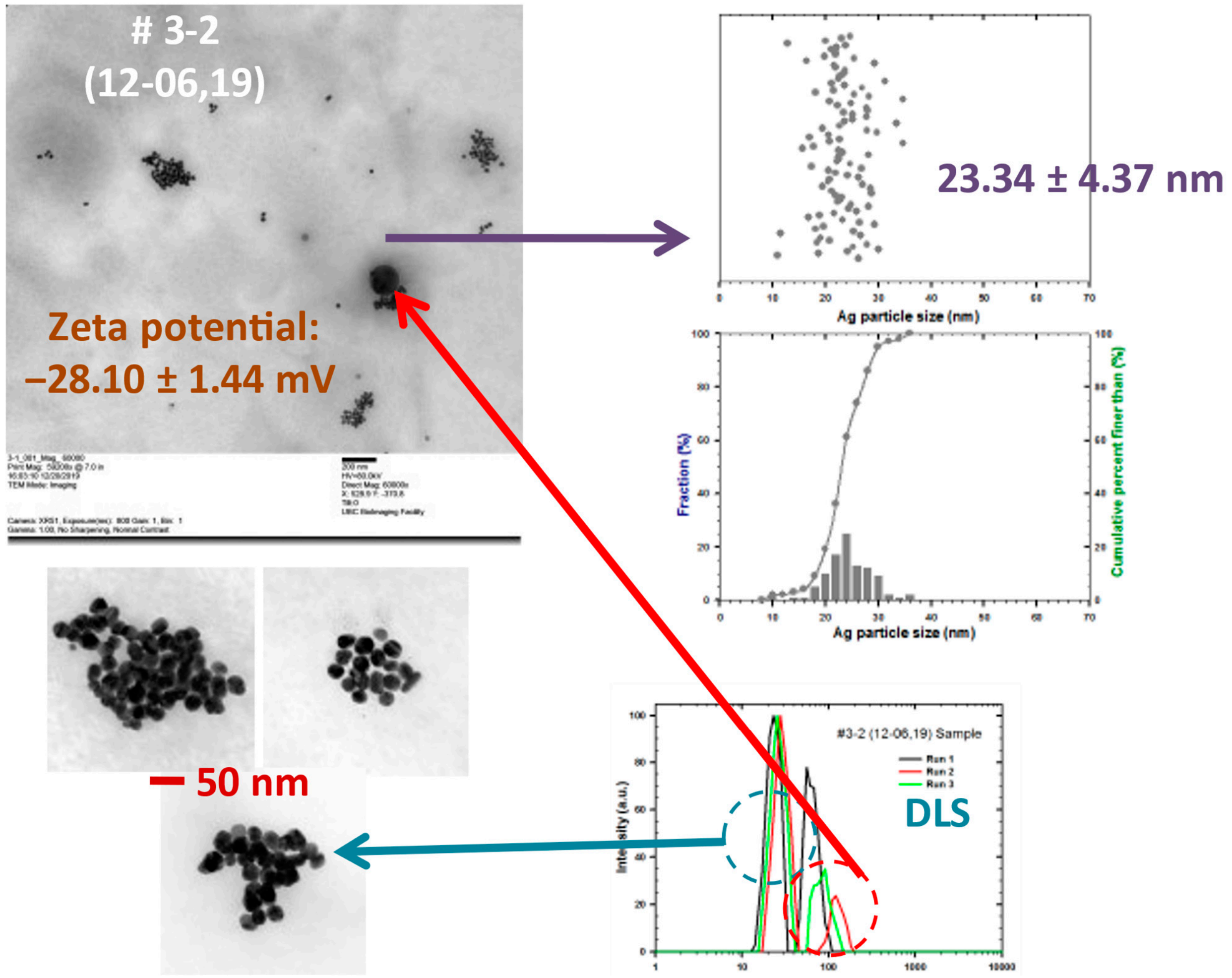

3-2
(12-06,19)
Zeta potential:
−28.10 ± 1.44 mV
23.34 ± 4.37 nm
Ag particle size (nm)
Fraction (%)
Cumulative percent finer than (%)
Ag particle size (nm)
50 nm
#3-2 (12-06,19) Sample
Run 1
Run 2
Run 3
DLS

3-2
(12-06,19)

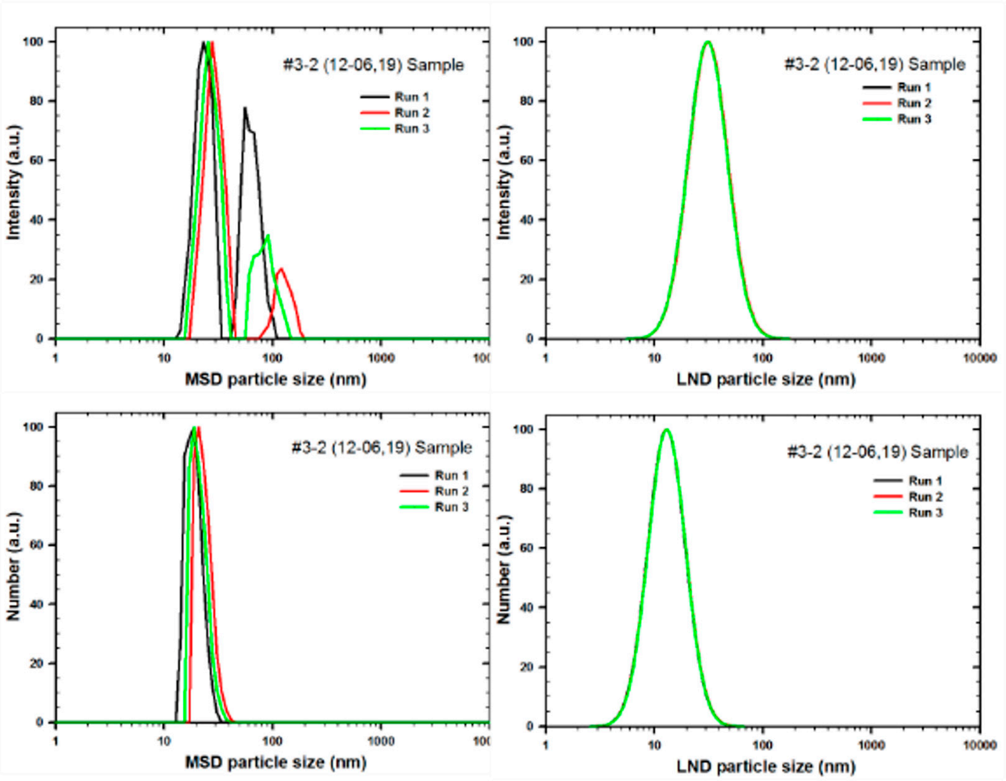

#3-2(12-06,19)	Aver. diameter[1]	Polydispersity index (PDI)	Aver. Diameter[2]
Run 1	31.05	0.192	19.05
Run 2	31.40	0.193	23.45
Run 3	31.06	0.191	21.31
**Aver.**	31.17±0.20	0.192±0.001	21.27±2.20

1: Calculated based on intensity-particle size distribution by software.
2: Calculated based on MSD/Number - particle size distribution

Supplementary Data-S1: Dynamic Light Scattering (DLS) graphs and size measurement of silver nanoparticles. The particle size distributions (MSD: multimodal size distribution, LND: lognormal size distribution) are expressed using intensity-based and numerical-based calculations.
